# A Review on Brittle Fracture Nanomechanics by All-Atom Simulations

**DOI:** 10.3390/nano9071050

**Published:** 2019-07-22

**Authors:** Sandeep P. Patil, Yousef Heider

**Affiliations:** 1 Institute of General Mechanics, RWTH Aachen University, Templergraben 64, 52062 Aachen, Germany; 2 Department of Civil Engineering and Engineering Mechanics, Columbia University, New York, NY 10027, USA

**Keywords:** fracture mechanics, molecular dynamics, brittle materials

## Abstract

Despite a wide range of current and potential applications, one primary concern of brittle materials is their sudden and swift collapse. This failure phenomenon exhibits an inability of the materials to sustain tension stresses in a predictable and reliable manner. However, advances in the field of fracture mechanics, especially at the nanoscale, have contributed to the understanding of the material response and failure nature to predict most of the potential dangers. In the following contribution, a comprehensive review is carried out on molecular dynamics (MD) simulations of brittle fracture, wherein the method provides new data and exciting insights into fracture mechanism that cannot be obtained easily from theories or experiments on other scales. In the present review, an abstract introduction to MD simulations, advantages, current limitations and their applications to a range of brittle fracture problems are presented. Additionally, a brief discussion highlights the theoretical background of the macroscopic techniques, such as Griffith’s criterion, crack tip opening displacement, J-integral and other criteria that can be linked to the fracture mechanical properties at the nanoscale. The main focus of the review is on the recent advances in fracture analysis of highly brittle materials, such as carbon nanotubes, graphene, silicon carbide, amorphous silica, calcium carbonate and silica aerogel at the nanoscale. These materials are presented here due to their extraordinary mechanical properties and a wide scope of applications. The underlying review grants a more extensive unravelling of the fracture behaviour and mechanical properties at the nanoscale of brittle materials.

## 1. Introduction

In the 21st century, fracture mechanics has been identified as one of the most emerging and promising fields of engineering mechanics. This is because the cracking-induced failure of devices and constructions presented a gigantic perturb to human communities, which is also related to safety and reliability concerns. But propitiously, advances in the field of fracture mechanics have helped to optimise many of the structural designs and, thus, to eliminate potential fracturing-related dangers and catastrophes [1]. In the last decades, fracture mechanics has been applied in a wide spectrum of fields, varying from railway construction [2], biology and medicine applications [3] to geophysics [4].

**Figure 1:**
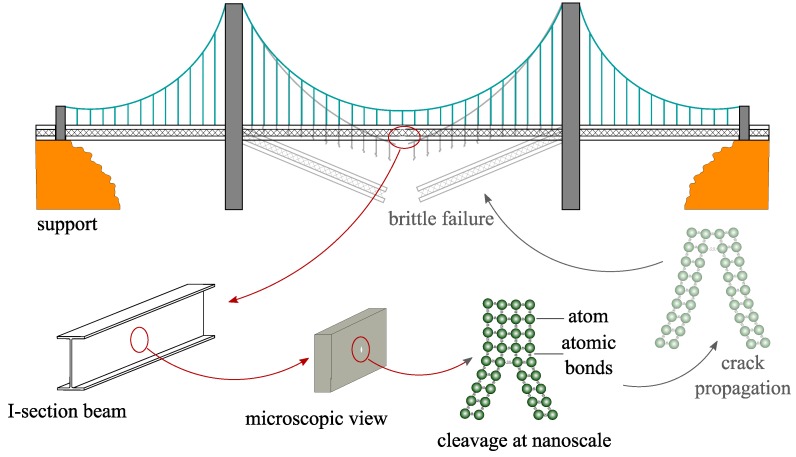
Schematic representation of a bridge and its possible collapse due to material fracture, which can be traced back to the collapse of atomic bonds on the nano scale.

In general, the fracture in materials usually initiates locally from a crack tip, which results in a global failure through crack propagation across the whole structure. In this connection, Figure 1 shows a schematic representation of a bridge, wherein a nano crack (cleavage) is initially presented. Due to the applied loading, high stresses occur at the nano crack tip, which might be followed by macroscopic crack propagation and potential collapse of the bridge. Therefore, understanding the mechanical response of the material under crack initiation and propagation at the atomic level is of great importance in fracture mechanics [5,6]. In this connection, multi-scale nano-macro analyses of brittle fracture that include scaling the atomic bond breaking forces have been introduced in, e.g., [7], where the forces next to the sharp crack tip and the atomic bond breaking process were addressed.

In brittle fracture, the materials exhibit little or no evidence of ductility or plastic degradation before the occurrence of the crack in the sense of material discontinuity [8,9]. Besides the fact that a fracture usually starts at a highly localised stress concentration area, such as at a welding notch due to lack of sidewall fusion, temperatures also affect the mechanical properties and, thus, have a considerable effect on the onset of brittle fracture [10]. For instance, if the temperature of steel is below its brittle-to-ductile transition temperature, then there is a higher probability that it will be susceptible to brittle fracture [11]. Moreover, the brittle fracture can also occur due to the fatigue and cyclic thermal loading [12].

Experiments play a vital role in new findings in science. The results obtained from experiments provide a basis for the understanding of mechanical processes, e.g., crack initiation, propagation and complete failure. However, as the growth of cracks is very fast in the event of brittle failure, the dynamics of crack initiation and growth is experimentally very challenging to study. When one observes the fracture in the structure at a macroscopic scale, it is a result of crack initiation and propagation at the nanoscale and carried over various length-scales [13]. Therefore, to fully understand any mechanism or process, experiments have to be coupled with computational models and theories. A traditional approach of modelling brittle fracture is continuum mechanics, in which the material is treated as a continuous mass neglecting the microscopic formation (e.g., chemistry, grain size, crystal structure, lattice spacing, etc.). Backed by powerful numerical solution schemes and energy-based fracture criterion methods, the continuum methods, and various other methods, have led to a comprehensive understanding of the brittle fracture mechanics as well as allowed to model fracture propagation at large scales. However, a failure mechanism which explains the loss of local material cohesion is not possible to describe using the macroscopic approaches [14]. In particular, during the crack opening at the atomic scale, the atoms in the crack tip region are pulled apart from each other as to unzip the brittle material (breaking of one atom-pair at a time).

In 1976, the molecular dynamics simulations were first time introduced to model fracture by Ashurst and Hoover [15]. The goal of that work was to study a few atoms system for fracture using a simple force-field. The small system was considered of the triangular lattice with 512 atoms, and the interactions were defined with linear snapping springs. Interestingly, Ashurst and Hoover referred to their numerical studies as “Newton’s laws”. However, after this work, it was widespread to use the words “molecular dynamics” to depict numerical studies of fracture at the atomic scale. From the late-1980s until now, a great interest has been shown by researchers on nanoscale fracture mechanics using molecular dynamics. For instance, Bitzek et al. [16] introduced a literature review as well as an overview of prospects of atomistic approaches for fracture modelling with a focus on crystalline materials.

Recently, Patil et al. [17] investigated the impact of crack length on the fracture properties, such as the fracture strength, toughness and strain energy release rate, using an all-atom model of 5.07 million atoms. In contrast to the finite element method (FEM), MD simulation nodes and elements (distance between nodes) are constructed not on an arbitrary basis, but more fundamental theories in chemistry, namely, the atoms as the nodes, the crystal lattice as the FEM mesh, and separated distances are based on the interatomic potentials. In contrast to FEM, atoms (nodes) and distance between atoms (elements) in MD simulations are not randomly defined but are governed by fundamental theories in chemistry. MD simulations provide a higher resolution in space and time of the fracture process compared to a continuum mechanics approach. Thus, nanoscale mechanics can be effectively investigated in detail by MD simulation. On the other hand, the system constitutes an enormous number of atoms, and it is essential to consider the interactions of several hundred to millions of atoms. Therefore, one may encounter situations where time and/or size constraints become indispensable. However, with the advancement of computer science and technology, MD simulations can nowadays be carried out on systems consisting of millions of atoms and simulation times up to one millisecond. Thus, in the recent years, MD simulations have been accentuated to study brittle fracture mechanisms of material at the nanoscale [18,19,20,21,22,23,24,25,26,27,28].

This review is organised as follows. Section 2 briefly describes the theoretical background of MD simulations with its advantages and current limitations. Although it does not aim to deepen into the theory, a summary of the method helps to get to the depth of all-atom simulations. In Section 3, a description of fracture mechanical properties and theoretical background of the macroscopic techniques that can be employed to estimate the fracture mechanical properties at the nanoscale are presented. Moreover, the critical milestones in MD simulations to study brittle fracture are summarised. The recent advances in commonly used highly brittle materials such as carbon nanotubes (CNTs), graphene, silicon carbide, amorphous silica, calcium carbonate and silica aerogel are presented in chronological sequence in Section 4. The tabular presentation includes a brief comparison among the different works mainly based on the size of simulation systems, force-fields and type of the considered brittle material. The conclusions are finally presented in the last section.

## 2. Principles of MD Simulation

MD simulation is a computational technique used to determine the time-dependent behaviour of molecular systems. In this method the initial coordinates and velocities of an ensemble of particles are provided as an input, this method then integrates the equation of motion and produce new positions and velocities at every iteration. For general techniques of molecular dynamics, we refer to the book of Rapaport [29]. In particular, MD simulation numerically solves Newton’s equations of classical motion,

(1) $$m_{i}\frac{\partial r_{i}}{\partial t^{2}}=F_{i},\;i=1,2,\ldots ,N.$$

Here, *N* is number of interacting atoms in the system. $r_{i}$ and $m_{i}$ represent the position and mass of atom *i*, respectively. $F_{i}$ is the force acting on atom *i*, which is experssed as

(2) $$F_{i}=-\nabla_{r_{i}}U\left(r\right).$$

An MD trajectory of the system is generated by updating the positions and velocities calculated as function of time. The expression for force-field can be divided into bonded and non-bonded interactions and its sum for all atoms give the total potential energy of the system U(r):

(3) $$U\left(r\right)=U_{\mathrm{bonded}}\left(r\right)+U_{\mathrm{non}-\mathrm{bonded}}\left(r\right).$$

### 2.1. Bonded Interactions

**Figure 2:**
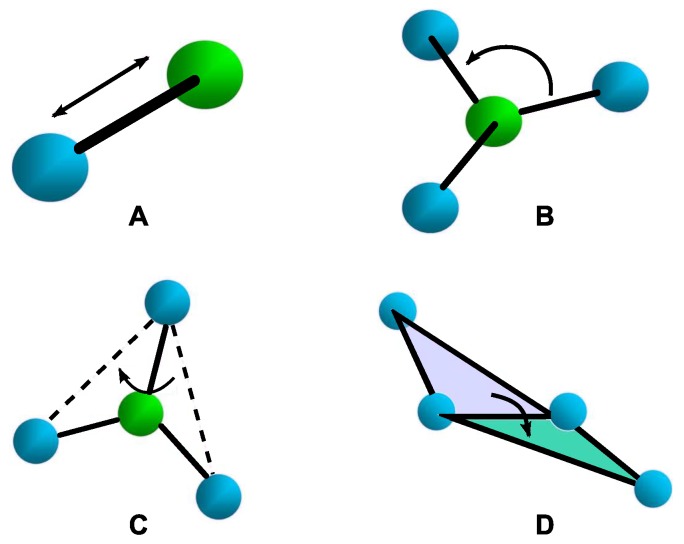
A schematic representation of covalent bondings, (**A**) bond-stretching; (**B**) angle-bending; (**C**) proper torsion; and (**D**) improper torsion.

Bonded interactions, $U_{\mathrm{bonded}}$ include covalent bond-stretching ($U_{\mathrm{stretching}}$), angle-bending ($U_{\mathrm{angles}}$), torsion potentials ($U_{\mathrm{torsion}}$), and out-of-plane improper torsion potentials ($U_{\mathrm{improper}}$) (see Figure 2). Covalent bonds are described by the first term in Equation (4). Bond stretching potential energy is modelled as Hookean spring ($U_{\mathrm{stretching}}$) with $K_{ri}$ denoting the spring constant, which represents the bond strength, and $r_{0}$ is the bond length at equillibrium (see Figure 2A). A bonded angle ($U_{\mathrm{angles}}$) term is described the second term in Equation (4) using three connected atoms system with force constant of $K_{\theta i}$ and an equilibrium angle of $\theta_{0}$ (see Figure 2B). Torsion potential is described by the third term in Equation (4) (see Figure 2C). Here, $V_{i}$ is the coefficient in the Fourier series, $\phi_{0}$ is the torsional angle at equilibrium, $\phi_{i}$ is the actual torsional angle, and *n* is the number of minima or maxima between 0 and 2π. The last expression in Equation (4) represents the improper torsion, wherein $K_{\xi_{i}}$ denotes a large spring constant, $\xi_{0}$ is the improper angle at equilibrium, and $\xi_{i}$ represents the actual improper angle, which is the deviation from planarity (see Figure 2D).

In particular, it holds

(4) $$\begin{array}{cc} U_{\mathrm{bonded}}\left(r\right) & =\;U_{\mathrm{stretching}}\left(r\right)+U_{\mathrm{angles}}\left(r\right)+U_{\mathrm{torsion}}\left(r\right)+U_{\mathrm{improper}}\left(r\right)\\ & =\sum_{\mathrm{stretching}\;i}K_{ri}{\left(r_{i}-r_{0}\right)}^{2}+\sum_{\mathrm{angles}\;i}K_{\theta i}{\left(\theta_{i}-\theta_{0}\right)}^{2}\\ & \;+\sum_{\mathrm{torsion}\;i}\frac{V_{i}}{2}\left[1+cos\left(n\phi_{i}-\phi_{0}\right)\right]+\sum_{\mathrm{improper}\;i}K_{\xi i}{\left(\xi_{i}-\xi_{0}\right)}^{2}. \end{array}$$

### 2.2. Non-Bonded Interactions

**Figure 3:**
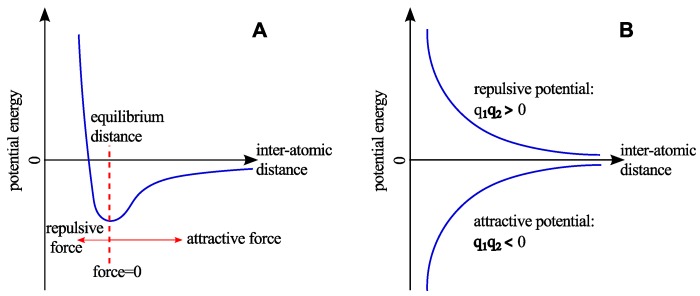
A schematic representation of the non-bonded interactions. (**A**) The Lennard-Jones potential shows the weak attraction and strong repulsion. (**B**) The Coulomb potential represents the interaction between two particles of same and opposite charges.

The non-bonded interactions, $U_{\mathrm{non}-\mathrm{bonded}}$ include Lennard-Jones and Coulombic interactions, that is

(5) $$U_{\mathrm{non}-\mathrm{bonded}}\left(r\right)=\sum_{i}\sum_{j}\left\{4\epsilon_{ij}\left[{\left(\frac{\sigma_{ij}}{r_{ij}}\right)}^{12}-{\left(\frac{\sigma_{ij}}{r_{ij}}\right)}^{6}\right]+\frac{q_{i}q_{j}}{4\pi \epsilon_{0}r_{ij}}\right\},$$

where the Lennard-Jones potential is represented by the first two terms of Equation (5), see, Figure 3A. Here, ϵ is the energy minimum, $\sigma =r/2^{1/6}$ is the distance between two atoms that gives the energy minima, and $r_{ij}$ is the distance between atom *i* and *j*. The last term of Equation (5) represents the electrostatic potential (Figure 3B), where $q_{i}$ and $q_{j}$ are the charges associated with the atoms *i* and *j*, respectively. Besides, $\epsilon_{0}$ is the permittivity of free space.

### 2.3. EAM Potentials for Metallic Atoms Interaction

The embedded atom model (EAM) potentials are presumably the most popular interatomic potentials for metals and alloys. It describes the metallic bonding characteristics more precisely than the two-body potentials [30,31,32]. This potential takes into account the effect of high electron density that presents all around each atom, and therefore, interactions of metal atoms near a free surface can be correctly modelled.

In the EAM, the potential energy *U* of a system consists of two parts, a pair interaction ϕ($r_{ij}$) between atoms *i* and *j* represents the repulsion between the electrostatic core–core and a cohesive term represents the energy of individual atoms *i*, which is always demonstrated through the effective electron density ρ at the location of an atom *i* by the function Φ($\rho_{i}$). The second term, Φ($\rho_{i}$), also describes as the energy of the ion core, and it depends on how much it is embedded in the electron sea. Thus,

(6) $$U\;=\;\sum_{i}\Phi \left(\rho_{i}\right)\;+\;\sum_{i<j}\phi \left(r_{ij}\right)\;.$$

ρ is evaluated by

(7) $$\rho_{i}\;=\;\sum_{j}\psi \left(r_{ij}\right),$$

where $\psi (r_{ij})$ is a electron density contribution from an atom *j*. Based on the experimental properties, the functions $\Phi (\rho_{i})$, $\phi (r_{ij})$ and $\psi (r_{ij})$ are altered in order to achieve the desired fit.

### 2.4. Other Force-Fields

In the literature, the Vashishta potential [33,34] has been used to represent the interatomic interaction for silica [17,35]. This potential can accurately replicate silica by incorporating both two- and three-body interactions. The potential energy is given by

(8) $$U_{ij}=\frac{H_{ij}}{r_{ij}^{\eta_{ij}}}+\frac{Z_{i}Z_{j}}{r_{ij}}exp\left(\frac{-r_{ij}}{r_{1s}}\right)-\frac{P_{ij}}{r_{ij}^{4}}exp\left(\frac{-r_{ij}}{r_{4s}}\right)\;,$$

(9) $$U_{ijk}=B_{jik}f\left(r_{ij},r_{ik}\right){\left(\cos\theta_{jik}-\cos{\tilde{\theta }}_{jik}\right)}^{2}\;,$$

where $r_{ij}$ represents the distance between *i*th and *j*th atom, $U_{ij}$ and $U_{ijk}$ represent the two- and three-body potentials, respectively. Also, $r_{1s}$ and $r_{4s}$ are the interaction cutoff distances. The first, second and third terms on the right-hand side of Equation (8) denote the steric repulsion, Coulombic interactions and electric polarisability, respectively. In Equation (9), $B_{jik}$ represents the strength of the three-body interaction. Also, $\theta_{jik}$ denotes the angle between the vectors $r_{ij}$ and $r_{ik}$, and ${\tilde{\theta }}_{jik}$ is constant. The parameter values for the above equations are available in Vashishta et al. [33,34].

The AIREBO force-field has been widely used to model the C–C interactions in carbon nanotubes and graphene [36,37]. In this force-field, the potential energy is given by

(10) $$U_{\mathrm{AIREBO}}\;=\;\frac{1}{2}\sum_{i}\;\sum_{j\neq i}\left[U_{ij}^{\mathrm{REBO}}\;+\;\sum_{k\neq i}\;\sum_{l\neq i,j,k}U_{kijl}^{\mathrm{TORSION}}\;+\;U_{ij}^{\mathrm{LJ}}\right]\;,$$

where $U_{\mathrm{AIREBO}}$ is the total potential energy of the system, $U_{ij}^{\mathrm{REBO}}$ is the REBO term, which describes potential between covalently bonded atoms, $U_{kijl}^{\mathrm{TORSION}}$ is the torsional part of the potential, which depends on the dihedral angle, and $U_{ij}^{\mathrm{LJ}}$ is the Lennard-Jones potential. One can note that this potential, used to study mechanical properties and fracture behaviour of carbon nanotubes and graphene materials, is with a modified cutoff length [24,38,39,40].

### 2.5. Numerical Solution of the Equations of Motion

In the literature, a number of numerical integration schemes have been proposed to solve Equation (1), and hence compute positions and velocities of every atom of the system as a function of time. One of the popularly used algorithms is leap-frog algorithm [41]. The leap-frog iterative formulae [41] is given by

(11) $$v_{i}\left(t+\frac{\Delta t}{2}\right)=v_{i}\left(t-\frac{\Delta t}{2}\right)+\frac{F_{i}\left(t\right)}{m_{i}}\Delta t,$$

(12) $$r_{i}\left(t+\Delta t\right)=r_{i}\left(t\right)+v_{i}\left(t+\frac{\Delta t}{2}\right)\Delta t.$$

In the leap-frog algorithm, velocities and positions are of the third order, and therefore, this numerical integration scheme is equivalent to the Verlet [42] algorithm.

### 2.6. Advantages and Limitations of MD Simulation of Fracture

MD simulations have been utilised to study dynamics properties at the nanoscale, e.g., viscosity, diffusivity, thermal conductivity and structural relaxation times. It has also been used to explore the physical properties of advanced nanostructured materials that do not exist or cannot presently be created. Due to high temporal and spatial resolution, MD simulations are ideally suitable to investigate the fracture behaviour of brittle materials, wherein crack initiation, propagation and complete failure occurs in picoseconds.

Nowadays, typical MD simulations can be performed on systems containing hundred thousands, or, even, millions of atoms, and for simulation times higher than one millisecond. However, to compare these numbers with macro and microscale systems, one may face situations, where time and/or size limitations become crucial. For example, in experiments related to dynamical processes, the relaxation times of the physical properties last for longer than the time scale of a few milliseconds. Thus, choosing MD system sizes with sufficient complexity and sufficiently long timescales becomes the primary challenge in MD simulations.

## 3. Fracture Mechanical Properties

Fracture is a failure process, characterised by an occurring discontinuity of a material as a result of subjected external loading or stresses. Generally speaking, it can be divided into two types, i.e., ductile and brittle fracture. On the one hand, ductile fracture involves substantial plastic deformation at crack edges before fracture. A typical example of ductile fracture under tensile deformation is the material necking, which leads to the formation of cavities, and cavities coalesce to form a crack. This class of cracks usually propagates slowly as well as in a stable manner, where a high amount of energy is dissipated before the ultimate failure. On the other hand, in the matter of brittle fracture, an insignificant amount of plastic deformation occurs before fracture. The material does not undergo necking, and cavities do not form. Moreover, cracks propagate rapidly, and very low amount of energy is dissipated in the form of plastic deformations before fracture. Cracks are usually unstable and propagate at high speed without any increase in the applied stresses. Thus, brittle fractures occur suddenly (without any warning), which is dangerous for any application.

Depending on the way of loading application, three different modes of material fracture can be distinguished:

1. Opening mode (Mode I): tensile stresses are applied perpendicular to the crack plane.
2. Shearing mode (Mode II): shear stresses act along the crack plane and normal to the crack front.
3. Tearing mode (Mode III): shear stresses act along the crack plane and parallel to the crack front.

Mode I is the common fracture mode, and it is used in the fracture toughness testing. Fracture toughness is the material property, which characterises the crack resistance offered by the material [43]. Mode II and III are both sliding (or shearing) modes, which are dominated by shear forces [44]. All these three modes of fracture are uncoupled in isotropic materials and coupled in anisotropic materials. The theories involved and the parameters used in fracture mechanics are discussed in detail by Anderson [43], Zhu and Joyce [45], and Sun and Jin [46].

In the literature, many theories have been presented to predict crack propagation in the materials. Here, the important criteria used at the nanoscale to calculate the fracture mechanical properties are discussed.

### 3.1. Griffith’s Criteria

In 1921, the English scientist A. A. Griffith proposed an energy-based investigation of cracks, which is regarded to be a major breakthrough in the discipline of fracture mechanics [47]. He performed experiments on glass specimens and developed an association between the crack sizes and the fracture stress. According to Griffith’s theory, the crack will propagate in the material when the released elastic strain energy of the system is greater than or equal to the energy needed to form new crack surfaces, i. e., surface energy. The proposed Griffith’s theory formula can be written as

(13) $$\sigma_{f}=\sqrt{\frac{2\;E\gamma_{s}}{\pi a}}\;,$$

where $\sigma_{f}$ is the rupture strength, *E* is the elastic modulus, $\gamma_{s}$ is the surface energy, and *a* is the crack depth. Griffith’s criterion is valid only for the linear elastic materials, which are ideal brittle materials like glass. This approach is also known as linear elastic fracture mechanics (LEFM) and the related theory is valid only for a plate of infinite size. Therefore, for a semi-infinite sized plate, Equation (13) becomes

(14) $$\sigma_{f}=\frac{1}{\alpha }\sqrt{\frac{2\;E\gamma_{s}}{\pi a}}\;,$$

where α is a geometric correction factor. In case of a double-edge notched specimen of semi-infinite size with crack height *h*, the correction factor α according to [48] reads

(15) $$\alpha =1.12\;+\;0.43\left[\frac{a}{h}\right]-4.79{\left[\frac{a}{h}\right]}^{2}+15.46{\left[\frac{a}{h}\right]}^{3}.$$

The concept of energy release rate was developed by Irwin [49], which was an extension of Griffith’s theory. The rate of change of elastic strain energy (potential energy) with the crack area reaches to its critical value $G_{c}$ when the fracture occurs. The strain energy release rate is expressed as

(16) $$G_{c}=\frac{\alpha^{2}\;\sigma_{f}^{2}\;\pi \;a}{E}\;.$$

Moreover, Irwin [49] proposed the critical stress intensity factor $K_{c}$, which can be described as fracture toughness of materials (material resistance to crack propagation). $G_{c}$ and $K_{c}$ are considered to be independent of size and geometry of the samples. In particular, the critical stress intensity factor was given as

(17) $$K_{c}=\alpha \;\sigma_{f}\sqrt{\pi \;a}\;=\;\sqrt{G_{c}\;E}\;.$$

Recently, many researchers have used the classical Griffith’s theory to investigate the brittle fracture behaviour of different materials in order to estimate $G_{c}$ and $K_{c}$ [18,19,20,21,22,23,24,25,26,27,28]. Recently, several works were carried out in MD simulations to estimate $G_{c}$ and $K_{c}$ and to understand the effect of the crack depth on the onset of crack, whereas comparisons with Griffith’s theory were carried out [22,50,51,52,53,54]. These studies provided two significant conclusions; First, for extremely small size crack, Griffith’s theory of fracture becomes inapplicable, and second, LEFM only can be used when the crack depth is at least four times of the material’s lattice constant.

### 3.2. Crack-Tip Opening Displacement (CTOD) Approach

Many engineering materials show inelastic behaviour under large loads, which cannot be modelled using the LEFM. Therefore, the crack-tip opening displacement (CTOD) theory was developed by Wells [55] to model the fracture, which occurs under large plastic deformations. Wells proposed the fracture toughness can be measured on the basis of opening at the crack tip. Due to the considerable amount of plastic deformation, crack tip loses its sharpness and becomes blunt, which gives rise to displacement at the crack tip. The effective crack length was considered to be $a\;+\;r_{y}$, where $r_{y}$ is displacement in the plastic zone of crack. The crack tip opening displacement δ for the effective crack length is given by,

(18) $$\delta \;=\;\frac{4}{\pi }\;\frac{G}{\sigma_{y}}\;=\;\frac{4}{\pi }\;\frac{K_{I}^{2}}{\sigma_{y}\;E}\;,$$

where *G* is the strain energy release rate, $\sigma_{y}$ is the yield stress, $K_{I}$ is the stress intensity factor for Mode I and *E* is Young’s modulus.

In 1960, Dugdale [56] introduced the strip-yield model, which was developed for calculating δ of the CTOD. For example, in an infinite plate, δ for the through-thickness crack with applied remote tensile stress σ from the strip-yield model is given by

(19) $$\delta \;=\;\frac{8\sigma_{y}a}{\pi E}\;\;\ln\;\;\sec\left(\frac{\pi }{2}\frac{\sigma }{\sigma_{y}}\right).$$

In the experimental work of Xia and Wang [57] and Kelling et al. [58], the plastic zone size and the fracture toughness have been determined using CTOD. There are some MD simulation studies in which the CTOD method has been utilised to calculate the strain energy release rate and the fracture toughness of the material [28,45,59].

### 3.3. J-Integral

Rice [60] introduced J-integral, which is applicable for fracture and crack analysis of nonlinear elastic materials as well as elasto-plastic materials. Rice proved that nonlinear strain energy release rate *J* is a line integral, which is path-independent. Hence, crack-tip stresses are characterised by J-integral in nonlinear elastic materials. *J* is defined as

(20) $$J\;=\;-\frac{dU}{dA}\;,$$

where *U* represents the potential energy, and *A* depicts the crack area. For the linear elastic material response, *J* is equal to *G*. *J* as a path-independent line integral is given by

(21) $$J\;=\;\int_{\Gamma }\left(w\;dy\;-\;T_{i}\frac{\partial u_{i}}{\partial x}ds\right)\;,$$

where *w*, $T_{i}$, $u_{i}$, Γ and ds are the strain energy density, components of traction vector, components of the displacement vector, boundary of territory containing the crack tip, and the length increment along the contour, respectively. The relationship between J-integral (*J*) and δ of the CTOD approach is given by

(22) $$J\;=\;m\;\sigma_{y}\;\delta \;,$$

where m represents a dimensionless constant, and it is dependent on stress state and material properties. Besides, $\sigma_{y}$ is the yield stress.

Recently, in the work of Cao et al. [61] for multilayered graphene oxide, the fracture toughness was measured using a J-integral method (a nonlinear fracture toughness analysis) and found to be ∼39 J/m${}^{2}$. The obtained value was at least two times or higher compared to the literature values. Other interesting studies of MD simulations, which used J-integral to compute fracture mechanical properties can be referred to in the literature as, e.g., Jin and Yuan [62], Nakatani et al. [63], and Khare et al. [64].

### 3.4. Other Criteria

In some cases, the classical approaches, such as the aforementioned ones, for modelling brittle fracture, can’t be applied. Therefore, essential non-classical methods can alternatively be employed. Neuber-Novozhilov criterion [65,66] is one of such non-classical approaches, which is used to model elastic plate fracture with angular and lune notches. This criterion is based on three concepts: First, the crack propagation problem can be treated as the equilibrium stability problem. Second, the length parameter is highly important in the description of crack propagation. Third, the average force per unit characteristic length in-front of the crack tip is greater than the local strength of the material. The criterion can be useful compared to Griffith’s approach, where the tip regions of arbitrary configuration and dimension, for any stress asymptotic, and generalised to problems with impact loads.

Griffith’s equation cannot be used in case of fracture of rubber since the deformation is nonlinear. Hence, Rivlin and Thomas [67] independently put forth an application of Griffith’s theory to rubber. Rubber undergoes a large elastic deformation except in a regime close to the crack tip. Rivlin and Thomas proposed that crack would propagate when the energy released by its propagation becomes equal to the tearing energy. On the other hand, tearing energy is equivalent to Irwin’s critical energy release rate, $G_{c}$ [68].

Kalthoff and Shockey [69] proposed a criterion for quasi-static fracture, which takes into account the time parameter, referred to as the Shockey-Kalthoff minimal time criterion. This criterion considers that rupture does not occur due to the momentary accumulation of stresses in the crack tip region, but it is a progressive process with respect to time.

### 3.5. Milestones in MD Simulations to Study Brittle Fracture

In Table 1, we present a timeline review of research works within nanoscale brittle fracture that led to significant advances in the last few decades. This includes a brief description of the MD systems, the applied fracture criteria as well as the significant results. This presentation shows how the MD simulations expanded over years to cover a wide variety of materials beside larger MD systems in connection with the advancements in computer capacities.

## 4. Brittle Materials—Advances in Fracture Study at Nanoscale

Under certain conditions, materials such as glass, ceramics, some types of polymers and metals can undergo brittle fracture. For instance, metals, that are generally ductile, might fail under very low temperatures in a brittle way, possibly with catastrophic consequences. In a mode-I brittle fracturing, cracks propagation path is nearly perpendicular to the applied loading, which creates a relatively flat surface at the fracture surface. Moreover, the fracture surface shows a typical pattern, e.g., some brittle materials are characterised by lines and ridges, which start at the fracture origin and spread along the crack surface. Due to the lack of plastic deformations prior to failure onset, this is considered as the most dangerous sort of fracturing. However, due to the remarkable thermo-mechanical properties of highly brittle materials, it is unavoidable to replace these materials for specific applications, e.g., silicon carbide used together with graphite for high-performance brake pad in a disc-pad braking system. Therefore, to use such high brittle materials, one has to understand its fracture behaviour and properties.

In this work, the considered brittle materials are the most studied for a wide range of current as well as potential applications by several researchers. For example, carbon nanotubes and graphene nanomaterials have attracted tremendous interest in research and industry due to their unique and outstanding electric and mechanical attributes. These materials have been used for many applications, e.g., candidates for the reinforcement in composite materials, transistors, battery electrodes, solar cells, and tissue engineering scaffolds. Another brittle material we have considered here is the amorphous silica (SiO${}_{2}$). Due to the excellent physical properties, it has been used in microelectronics, chromatography, chips, optical fibres, telescope glasses, nanoelectronics and nanopore sensors. Moreover, the other brittle materials, which we have reviewed here, are silicon carbide, calcium carbonate and silica aerogel. These materials have tremendous current as well as potential applications in the engineering field. There are many other impressive materials (e.g., silicon nitride and gold) that studied extensively in the literature. However, we did not consider those materials in this work due to the manuscript-length.

### 4.1. Carbon Nanotube

Carbon nanotubes (CNTs) have attracted a lot of scientific works in nanotechnology since their first observation [87]. They show remarkable properties due to their peculiar cylindrical nanostructure. The covalent chemical bonding of entirely sp-2 hybridised carbon atoms and smooth continuous hexagonal network result in the unique mechanical, electronic and thermal properties, which are far better than any current, comparable material and thus valuable in different fields, like in materials science, optics, and electronics. Walls of carbon nanotubes are one-atom-thick sheets of graphene, which are rolled at a certain chiral angle. The high mechanical strength of carbon nanotubes and low density together opened the door for a wide range of future applications. For instance, CNTs find numerous applications in composite materials. With the development of MD and advances in computational abilities, the new material composites and their properties can be analysed faster than never before. In the review by Thostenson et al. [88], the developments in the research on CNTs and their composites were covered until 2001.

A single-walled carbon nanotube (SWCNT) can be imagined as rolling of a graphene sheet to a smooth tube with a fixed diameter. In the present work, we modelled the SWCNT of (20,20) with the length of 20 nm, which consisted of 6560 atoms. Moreover, the double-walled carbon nanotube (DWCNT) was modelled with (20,20) outer CNT and inner CNT of (15,15), which has 11480 atoms. The atomistic simulations were implemented in the open-source package LAMMPS (Large-scale Atomic/Molecular Massively Parallel Simulator) [89]. The intratube and C–C atoms short-range interactions were defined by Brenner’s second-generation reactive empirical bond-order (REBO) force-field [36,37], whereas the van der Waals force was considered to represent the carbon atoms’ long-range interactions. Moreover, the tensile tests were implemented to the centre cracked SWCNTs and DWCNTs.

**Figure 4:**
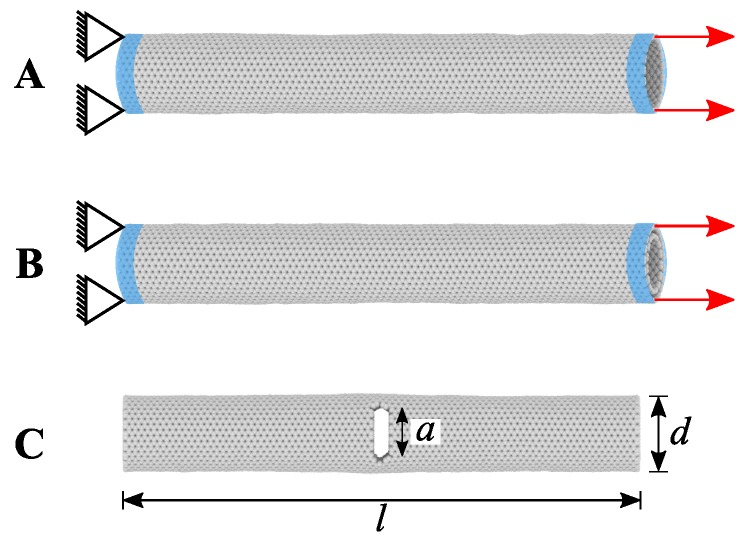
MD simulation setup: Schematic representation of (**A**) SWCNT and (**B**) DWCNT pulling. (**C**) Describes the dimensions of cracked CNT, where *l*, *d* and *a* represent the length, diameter and crack length, respectively. All the models were fixed at one end and pulled at another end.

**Figure 5:**
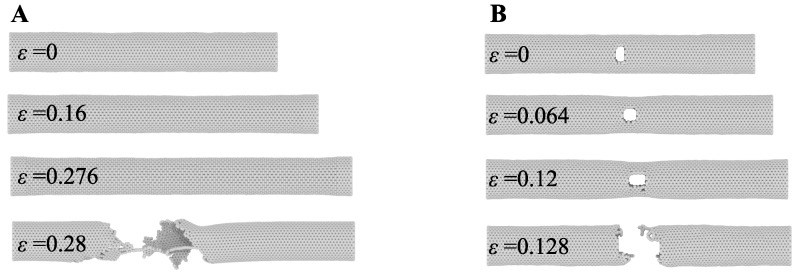
The snapshots show the fracture of (**A**) the SWCNT without crack and (**B**) the cracked SWCNT under constant pulling velocity.

Figure 4 shows the model setup. In the MD systems, initial velocities were assigned from the Maxwell-Boltzmann distribution [90] and then followed by energy minimisation using the conjugate gradient method at a given temperature. Subsequently, each MD simulation system was equilibrated for 100 ps using NVE ensemble to reach a steady state. The relaxed model of CNT was then pulled under NVT conditions at one end with a constant velocity of 0.2 Åps${}^{-1}$, while the model was fixed at another end. In case of the pre-defined crack models, after initial relaxation, central parts of the CNTs with specific crack lengths (block of three-rows of atoms and circle at the end) were removed by deleting atoms (see Figure 4C). The pre-defined crack models were energy minimised and equilibrated before the pulling simulations. Figure 5 shows the crack propagation in SWCNT with and without pre-defined crack. For both models, up to the strain of 0.276 (without crack) and 0.12 (with centre *a* = 1.2 nm), there was no crack propagation observed. However, in the next ϵ < 0.008 the crack initiated and sudden fracture occurred. Therefore, the modelled SWCNTs and DWCNTs feature the mechanical properties of brittle material.

**Figure 6:**
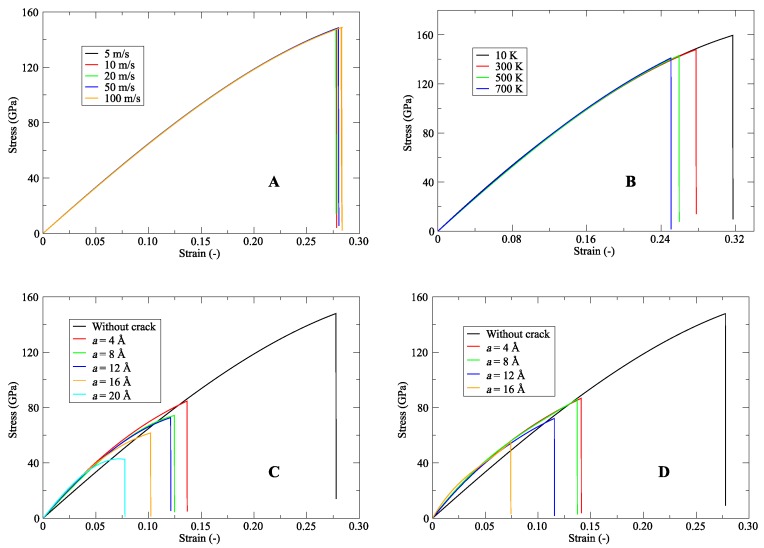
Stress–strain behaviour for (**A**) a range of pulling speed, and (**B**) change in temperature of the (20,20) SWCNT. Moreover, stress–strain trajectories for various crack depths of (**C**) SWCNT and (**D**) DWCNT.

Figure 6 shows the stress–strain trajectories of SWCNTs and DWCNTs. Here, stresses in the loading direction are calculated from the energy-strain curves. In this work, a wide range of pulling speeds was considered, and it was found that pulling speed has no significant influence on the stress–strain curve (see Figure 6A). However, temperature presents an important factor in the mechanical characteristics of CNTs. As temperature increases, the fracture stress and strain decrease significantly (see Figure 6B). The obtained trends for the variation of the parameters were analogous to those found in Wei et al. [91]. To figure out the effect of the crack on the stress–strain response of CNTs, the centre crack of length was varied from no crack to 20 Å and no crack to 16 Å for SWCNTs and DWCNTs, respectively. Figure 6C,D show the stress-strain relationship in the loading direction. All these curves were nearly linear until the onset of the fracture. As *a* increases, the fracture stress and strain decrease significantly. In all the parameter variations as well as a change in crack length simulations, the elastic modulus was nearly constant (0.78 ± 0.03 TPa).

### 4.2. Literature Review of Carbon Nanotubes: Past and Recent Advances

In 1991, Iijima [87] reported the possible production of needle-like carbon finite tubes, which was the first evidence of CNTs existence. Arc Discharge Evaporation Method was used for CNT production, and the nanostructure of the CNT was described using electron microscopy. Yakobson et al. [92] performed MD simulations of SWCNTs and DWCNTs of different helicities, wherein interatomic forces described by Tersoff-Brenner potential. It is found that CNTs have enormous breaking strains, and these breaking strains decrease slightly with increasing temperatures.

One of the first studies on the fracture of CNT was carried out by Belytschko et al. [38] in 2002. The fracture of a zigzag nanotube was studied using a modified Morse potential, in which a bond angle-bending was added. Moreover, the influence of cutoff on fracture behaviour was studied. The following are the primary conclusions of the work,

■ the scope of collapse strains of defect-free CNTs was in the range of 10 to 16%,
■ a decrease in the strength was obtained in connection with defect occurrence,
■ fracture strain showed strong dependency on the inflection threshold in the potential, and
■ chirality seemed to have just a moderate influence on CNTs strength.

Li and Chou [93] explored the relations between sectional stiffness attributes and constants of the force-field using a simple harmonic form of energy. A link between structural mechanics and MD was established, where they found that by increasing the tube radius, a monotonic increase of Young’s moduli of zigzag CNTs and armchair is obtained. The fracture of the multi-walled CNTs (MWCNTs) was investigated by Liew et al. [94]. Interatomic forces were modelled using the covalent bonding interactions, and the interlayer interaction was characterised with the long-range van der Waals potential. They found that for the MWCNTs, cracking starts at the outermost layers. Besides, the stress–strain curves were extracted to describe the elasto-plastic behaviour of CNTs.

Buehler et al. [95] suggested a mesoscopic framework for SWCNTs to study self-assembly and behaviour under compression, bending and tension. The proposed mesoscopic model was able to describe the self-assembly mechanisms and the mechanical responses of CNTs. Moreover, the self-folded structure of CNT was remarkably stable and could be used as a sensor for many chemical surroundings. Furthermore, under bending, the fracture-related attributes governed the behaviour of a CNT bundle. Han et al. [96] carried out MD simulations on polymer/CNT composites models, and figured out an increase in CNTs elastic modulus at lower strains. Nikiforov et al. [97] investigated the strain release in a wave-like distortion mode for armchair SWCNTs and MWCNTs, where for MWCNTs, nearly no hysteresis between bending and unbending was observed. Neyts et al. [98] performed MD and Monte Carlo (MC) simulations considering adding carbon to the cluster, and revealed for the first time the simulated growth of an armchair SWCNT. Vijayaraghavan et al. [99] found that the existence of bends has a significant influence on the elastic behaviour of SWCNTs. Due to these imperfections, changes in the elastic properties were highly influential to the length of the CNTs. However, these variations were negligible at high temperature. Li et al. [100] studied the strain rate, geometrical dimensions and temperature dependent a ductile or a brittle failure mode of CNTs at the junction using AIREBO potential with 0.246 nm cutoff. Moreover, twist and chirality modes play an influential role in the deformation.

In 2016, Yang et al. [101] computed the toughness at the nanoscale using COMPASS force-field with a maximum permitted bonding force of 10 nN. It was concluded that stresses around the atomic defects were nonuniform, and the propagation of fracture was impulsive and continuous. CNT-reinforced nano-composites have been studied using MD simulations to analyse the effect of CNT presence on the mechanical behaviour of nano-composites [102,103]. Hossain et al. [104] investigated the elastic and fracture properties of CNTs using Stillinger–Weber (SW) and AIREBO potentials, and found that SW potential was computationally less costly as well as exhibit reliable for extreme mechanical behaviour. Li et al. [105] investigated MD models of a pure polymer matrix as well as CNT/Epoxy composite. CNT/Epoxy composites showed an increase of about 24.8% of the tensile strength and 34.3% of the elongation at the breakage point. Moreover, van der Waals forces of CNTs seemed to play an important role in the fracture response. Furthermore, the J-integral expressions were used, where an increased in the energy release rate of 35.7% was obtained. Recently, Wang et al. [106] studied the mechanical performance of carbon nanotube films subjected to ballistic impact using MD simulations. The films showed excellent mechanical resistance, due to brittle cracking of the individual nanotubes combined with a ductile collapse (e.g. delamination or slipping) at the interfaces between the tubes.

### 4.3. Graphene

Theoretical studies on graphene can be found in the literature since 1947. However, it was isolated and characterised by Novoselov et al. in 2004 [107,108] at the University of Manchester. Graphene is an allotrope of carbon, an atom-thick sheet of carbon atoms in a honeycomb formation. It is the first pure two-dimensional material that has ever existed. Graphene has several excellent properties, which make it a material of interest in various fields and has a broad range of applications. It has a high thermal as well as electrical conductivity, high electron mobility, and low affinity towards the absorption of light. Graphene has unique mechanical properties, e.g., low density, high elasticity modulus, high surface-to-volume ratio, etc. These mechanical properties also influence other properties of graphene, e.g., structural changes due to mechanical deformation also change the electronic properties of graphene. Thus, these properties should be thoroughly studied to maximise the potential applications of graphene.

**Figure 7:**
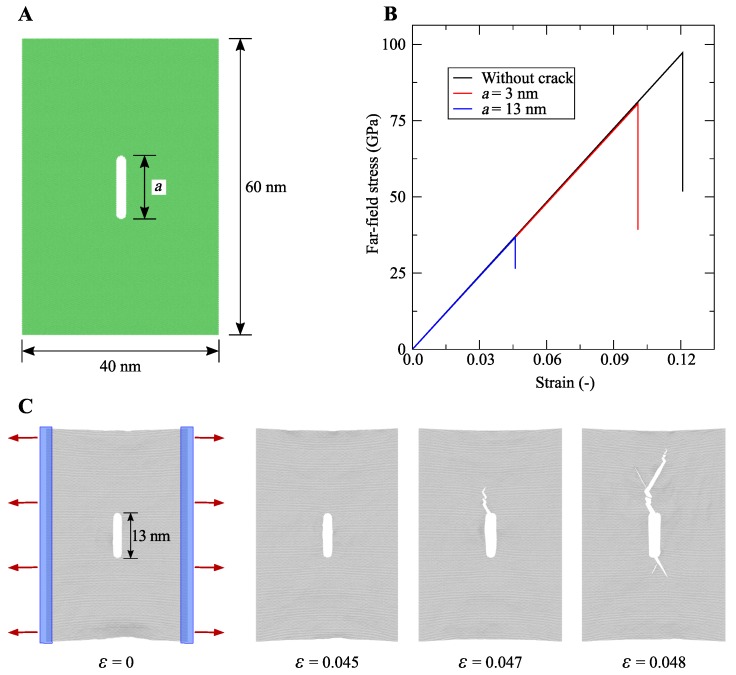
(**A**) An all-atom model of a monolayer graphene sheet under tension with a pre-defined central crack. (**B**) Stress–strain trajectories during brittle fracture. (**C**) The model was pulled on both sides with a constant strain rate, such that pulling directions are perpendicular to the crack depth, which is marked by red arrows. Snapshots illustrating the fracture propagation at subsequent values of the strain.

In the following, a graphene sheet is modelled using Visual Molecular Dynamics (VMD), and coordinates of the atom coordinates are retrieved [109]. The dimensions of the armchair graphene sheet are 60 × 40 nm${}^{2}$, which contains 92584 carbon atoms. The centre crack of different lengths is modelled, and LAMMPS is used to run these simulations. In case of the pre-defined crack models, after initial relaxation, central parts of the graphene sheet with specific crack lengths (block of three-rows of atoms and circle at the end) were removed by deleting atoms. The pre-defined crack models were energy minimised and equilibrated before the pulling simulations. The interactions between the C–C atoms are modelled following Brenner’s 2nd-generation reactive empirical bond-order (REBO) force-field [36,37]. The cutoff parameter for REBO is kept as 2.0 Å. The system is relaxed with a time increment of 0.0005 ps for 50 ns. After relaxation, the graphene sheet is pulled at a constant strain rate of 0.001 ps${}^{-1}$. Figure 7A illustrates the atomic structure of a graphene sheet with dimensions.

Figure 7B presents the stress–strain trajectories of armchair graphene for different pre-defined crack lengths at 300 K. The results reveal that as the crack length increases the fracture strength and failure strain significantly decrease. The snapshots in Figure 7C show the progress of the fracture.

### 4.4. Literature Review of Graphene: Past and Recent Advances

We focus in the following on the fracture characteristics of graphene. Though it has a high Young’s modulus, its fracture is characterised to be brittle, as it is likely to crack like ceramic materials. These properties have been investigated using MD simulations in several research works as briefly described in the following.

In 2007, Khare et al. [64] coupled QM and MD methods to derive of energy release rate by J–Integral method using Tersoff Brenner potential. They detected only a slight dependency between the strength of CNTs and the shape of defects, and the obtained fracture strengths showed a reasonable agreement with Griffith’s formula in a finite graphene sheet. Zhao et al. [39] studied the influences of the size and chirality on the elastic response of graphene nanoribbons applying the AIREBO potential with a cutoff set to 0.2 nm. They could extract the elasticity modulus, Poisson’s ratio, the fracture strength and the related strain for bulk graphene. Moreover, in the uni-directional tensile test, the fracture strength and strain were higher for the zigzag graphene compared to the armchair graphene. The anisotropic mechanical characteristic of graphene sheets was confirmed by Ni et al. [110]. The resultant Young’s moduli were found to be 1.13 and 1.05 TPa for the longitudinal and transverse modes, respectively.

Zhao et al. [111] proposed the quantised fracture mechanics theory, wherein the relationship between the fracture strength and the related crack length was described. Moreover, for temperature ranges between 330 and 2400 K, the values of Young’s modulus was less influenced. However, the fracture strength at a temperature of 2400 K was around 40% of the strength at 300 K. Pei et al. [112] studied the attributes of functionalised graphene, such as the position, distribution and coverage of the functional radicals. Min et al. [113] investigated the shear properties of a graphene sheet of size 10.08 × 10.22 nm${}^{2}$. It was observed that increasing the temperature up to 800 K leads to shear modulus increase, and then it starts to decrease for higher temperatures. However, shear strength and cracking strain decrease as the temperature increases. Wang et al. [114] reported the role of mechanical strain and temperature in the formation of SW defects. It was found that at low temperatures, a strain can only lead to brittle fracture via bond breaking. Xu et al. [115] investigated fracture in graphene using quantum mechanics and continuum mechanics, wherein the crack growth mechanism and the critical stress intensity factors values have been proposed.

Zhang et al. [116] studied square-shaped zigzag as well as armchair graphene with a side length of 20 nm. The investigation showed that both the fracture strength and elasticity modulus decrease with the increasing of acetylenic linkages, and fracture is initiated in graphene at the low atomic density and in the acetylenic linkages at weak single bonds. A similar study has been done by Yang et al. [117], wherein it is observed that the increase in the number of acetylene groups, the obtained fracture stress decreases gradually with armchair case and remains nearly unchanged with the zigzag case. Zhang et al. [118] studied fracture process dependency on defect configuration, temperature, and strain rate. An interesting study was also introduced by Ansari et al. [119], wherein MD simulations results were found to agree with those calculated by non-local elasticity plate models. Dewapriya et al. [40] simulated graphene sheet with 1008 carbon atoms using AIREBO potential. It was concluded that vacancy defects and thermal effects have a significant influence on the fracture strength, and the energy dissipation rate was proportional to the strength. Moreover, having random vacancies in graphene leads to a singular stress field, which is similar to macroscopic fracture mechanics.

The classical Griffith’s theory of brittle fracture applied to graphene was verified using experiment and MD simulations by Zhang et al. [23]. The results showed that the fracture strength was the lowest for the zigzag shape and the highest for the armchair shape. Moreover, the fracture toughness was measured as the critical stress intensity factor. Yin et al. [25] also compared MD simulation results of graphene with Griffith criterion and ended up with numerical results, which were in line with that based on Griffith’s criterion. Rajasekaran et al. [120] investigated vacancy defects, and found that the mechanical properties and failure morphology of graphene were altered significantly. Shekhawat and Ritchie [121] reported an unravelling of the grain-size-dependent variations in strength and toughness of nanocrystalline graphene by using a combination of continuum modelling, weakest-link statistics and large-scale MD simulations. Qin et al. [122] studied the influence of topological defects in rippled graphene. The investigation concluded that an inverse proportionality exists between the Poisson’s ratio of rippled graphene and the aspect ratio, whereas negative Poisson’s ratios were observed for aspect ratios greater than 0.066. Winczewski et al. [123] performed modelling and simulations of penta-graphene, and found that tersoff potential most correctly describes penta-graphene.

Recently, determination of reinforcement effects was studied for graphene-polymer [124], single- layered graphene-coated polyethylene [125], and graphene-reinforced silica aerogel nanocomposites with single and multiple layers [126]. In these nano-composite studies, graphene has proven to be an extraordinary reinforcing candidate for the matrices, wherein fracture of graphene is investigated in detailed. Moreover, Hansen-Dörr et al. [127] reported the effect of point defects in graphene on the critical stress intensity factor and elastic properties using MD simulations.

### 4.5. Silicon Carbide

Silicon carbide (SiC) is also known as Carborundum. It can be mass produced and mainly used as an abrasive. It has a lot of potential applications such that involving high-frequency radiation hardened and high-temperatures. It also finds numerous applications in the fields of electronics, automobile parts, foundry and jewellery. It has recently been used to synthesise graphene by graphitisation at high temperatures [128]. In the review by Rountree et al. [129], developments in the molecular dynamics simulations in the crack propagation in brittle materials until 2002 were discussed.

### 4.6. Literature Review of Silicon Carbide: Past and Recent Advances

A literature review of recent and past research works that focused on studying the brittle nature of SiC using MD simulations is presented in the following. Briefly, we also present a description of the MD systems, the fracture criteria and some significant results, which allows us to track the advancements in this field.

Szlufarska et al. [130] reported a ring technique to study the nanoindentation damage and found that the crystalline to amorphous transition occurred due to the dynamics of dislocation loops and defect stimulated growth. In an extension of this work, it was observed that damage in amorphous SiC exhibits lower damping and it is spatially less localised than in 3C-SiC. Cracks propagation were observed differently for different crack propagation directions of 3C-SiC crystal by Kikuchi et al. [19] using modified SW potential. The system was stretched gradually up to the almost critical strain and concluded that cleavages in [110], [001], and [111] fracture directions are unstable against branching and slip, respectively. In case of a crack tip reconstruction, crack propagation at low-speed is unstable in [111] cleavage plane [131]. However, for at high-speeds of crack propagation, [110] cleavage plane turns to be unstable and defects on to [111] planes [131].

Pan et al. [132] did interesting work on brittle properties of SiC nanotubes using tersoff potential, wherein modified short-range interactions for the system size of 4896–17136 number of atoms were used. There have been many research work shade a light on the nanowire application of 3C-SiC [133,134]. The large plastic deformations and brittle behaviours of 3C-SiC were investigated by Wang et al. [134]. It is found that due to microstructural anisotropy, high variation in mechanical properties was observed, and microstructures strongly influenced the mechanical behaviours and properties. Pizzagalli et al. [135] improved the capability of the SW potential to describe defects and plastic characteristics of silicon; however, there was the limited success of the new parameters to elucidate fracture. MD simulations have been effectively used in the wear [136] and cutting [137,138,139] processes. Xiao et al. [137] observed the transition of brittle–ductile cutting mode. Moreover, they found that brittle fracture was occurred due to the presence of tensile stress around the cutting zone, and initiation of crack and propagation direction varies with undeformed chip thicknesses. Liu et al. [140] studied the scratching process of SiC at the nanoscale at various scratching speeds, and found that for high scratching speed, generates more amorphous structure atoms and high temperature.

Recently, Li et al. [141] investigated shock-induced defects and fracturing in 3C-SiC at an initially high temperature of 2000 K and a high rate of the tensile strain of ∼10${}^{10}$ s${}^{-1}$. This work concluded that due high temperature, the spall fracture strength was reduced, which was around 33% less than strengths deduced at 300 K.

### 4.7. Amorphous Silica

Silica (or silicon dioxide) has the chemical formula SiO${}_{2}$. It is one of the most abundantly found materials on earth, as usually found in nature in the form of sand or quartz. In the computational studies, amorphous silica can be prepared from the crystalline silica by applying an annealing process. From the earliest days of history to the modern era, amorphous silica has been an essential material in various applications, such as glass, wafer, electronic devices, and nanotechnological devices. In this, MD simulations presented a powerful tool to investigate the structure–property relationship of amorphous silica. In work by Muralidharan et al. [142], the progress of the investigation of brittle fracture of vitreous silica via MD simulations was reviewed until 2005.

### 4.8. Literature Review of Amorphous Silica: Past and Recent Advances

In the following, critical milestones in the MD simulations of brittle fracture behaviour of amorphous silica are reviewed. This chronological sequence presentation, together with the description of the MD systems and the applied fracture setups allows us to compare and follow the advancements in this research area.

Ochoa et al. [143] conducted one of the first studies on the formation of amorphous silica using simplified Born-Mayer-Huggin potential, wherein crystalline silica was heated and quenched in three steps, and at the end, a solid glass was obtained. Nakano et al. [144] performed MD simulations of the systems of 1.12 million atoms considering a wide range of the densities. For the low-density of 1.4 g/cm${}^{3}$, the growth of the largest pore resulted in a complete fracture. The roughness exponent of the occurring fracture edges well agreed with the experimental values. Another interesting study on amorphous silica, which contained a million atom, was performed by van Brutzel et al. [145]. The important finding of this work was the multiple branches that were observed at advancing crack fronts, and a strain energy dissipation was deduced during creating of nanoscale pores. Pedone et al. [146] developed pairwise empirical potentials, wherein silica was obtained from the process of melt-quench of β-cristobalite. In this, negative values of Poisson’s ratio were reported for α-cristobalite, and because of the sample deformation, a α-β phase transformation occurred. Moreover, the enforcement of a zero Poisson’s ratio resulted in brittle fracturing. Rountree et al. [147] described the fracture process occurred in amorphous silica. Due to crack nucleation, cavities up to 20 nm were observed ahead of the crack tip. These cavities, coalesce and merging during crack propagation resulted in mechanical failure.

Vashishta et al. [148] proposed an effective interatomic potential for MD simulations and applied the potential to multi-million atom MD simulations of cracking, indentation and impact-induced damage. In the literature, amorphous silica has been studied for nucleation of defects at high strains using BKS potential [149] (the potential suggested by van Beest, Kramer, and van Santen) [150,151]. A new mechanism for damage cavity nucleation was revealed via strain-induced defect transport by Nomura et al. [152], wherein crack nanocolumns were produced due to nanocavities coalesce. Castro et al. [153] investigated crack initiation and propagation due to complex chemical reactions. Yuan et al. [154] studied the densified glass samples, which are prepared using constant hydrostatic pressure during the melt-quenching process. The defects in the samples promoted the shear flow and, thus, provided a further means for dissipation of energy. A detailed study on the annealing process, which is required to prepare amorphous silica from the crystalline silica, was conducted by Chowdhury et al. [155]. It was found that the cooling rate and the related temperature significantly change the silica structure. At high strain rates, complete failure occurred in the volume model, however without localisation of any damage.

Recently, Rimsza et al. [156] reported the behaviour of an atomistic amorphous silica model with a slit crack under Mode I stress (far–field loading). The J–integral around the crack tip was evaluated by an Irving–Kirkwood scheme with a Lagrangian kernel estimation [157]. The resulting $K_{IC}$, 0.76 ± 0.16 MPam${}^{1/2}$, were in good agreement with experimental values. Moreover, an inelastic region of 3 nm in diameter was noticed from the stress fields and dissipation energies. In silica glass, Chowdhury et al. [158] investigated the fracture mechanisms and the influence of surface crack on the mechanical attributes, where they figured out how the strength of fracture is greatly affected by the fracturing.

### 4.9. Calcium Carbonate

In recent years, brittle ceramic materials have been in focus of active researchers in order to get highly reliable structural elements for engineering applications. In particular, ceramic has the ability to sustain much higher temperatures compared to metals. Therefore, it can be used in many applications with elevated operating temperatures. Besides, due to its low cost and long life, ceramic can replace metallic materials.

In our previous work, MD simulations were applied to understand the brittle fracture behaviour of aragonite nacre (CaCO${}_{3}$) [27]. These simulations concluded that there are two regimes for fracture: First, if the depth of flaws smaller than 1.2 nm, CaCO${}_{3}$ remains flaw-insensitive. Second, having larger flaws yields a behaviour that can be well-described within the macroscopic Griffiths fracture approach. Moreover, our MD fracture simulations of flawed CaCO${}_{3}$ showed a good agreement in the stress distribution between the MD results and the results of finite element analysis applied to the same problem [27]. Moreover, a bottom-up approach was proposed to study the brittle fracture of CaCO${}_{3}$, which supplied a fundamental understanding of the multiscale modelling of the cracking process [26]. The brittle crack propagation at the nanoscale was modelled using the MD technique. The phase-field modelling (PFM) parameters were extracted from the MD simulations and then applied in the higher-scale macroscopic model. This bottom-up scheme enabled us to give the PFM parameters to complete physical meanings. Moreover, the new material design could be eased using this combined approach.

**Figure 8:**
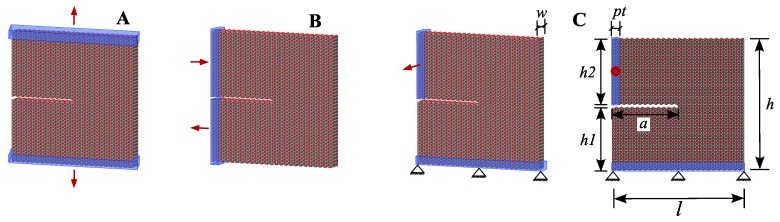
The three fracture modes of all-atom models of aragonite tablets. (**A**) Schematic representation of the perspective view of Mode I fracture. The model was pulled on both sides with constant velocities, such that pulling directions are perpendicular to the crack depth, which is marked by red arrows. (**B**) The Mode II model was loaded parallel to the crack surfaces with pushing the top and pulling bottom portion of the model. (**C**) In the Mode III, or tearing mode, the top portion of the model was loaded parallel to the crack front the crack surfaces, in which bottom portion was fixed. The dimension *a* denotes the crack depth, while *h*, h1, h2, *l*, *w* and pt represent the total height, bottom part height, top part height, length, width and pulling layer thickness, respectively.

Following this, an edge-crack aragonite tablet model was built, and it was used for three different loading conditions to represent three modes. The model was constructed with length (*l*), width (*w*) and height (*h*) of 19.80 × 2.20 × 19.70 nm${}^{3}$, with lower height (h1), upper height (h2) and crack depth of 9.40 nm, 9.90 nm and 9.96 nm, respectively. For all cases, a pulling layer was considered with thicknesses (pt) of three atomic layers wide. In the Mode I, the aragonite tablet under constant velocity pulling on both sides, which refers to the applied tensile loading perpendicular to the plane of the crack (Figure 8A). For the Mode II, constant pull and push velocities applied parallel to the crack edges and normal to the crack front (Figure 8B). In the Mode III, the bottom part was fixed, and a constant velocity was applied on the upper part of the model, i.e., the applied load was acting parallel to both the plane and front of the crack (Figure 8C).

For the following MD results, the MD package GROMACS 5.0.4 [159] was used. For aragonite interactions, a CaCO${}_{3}$ force-field, introduced in [27], was applied, which took into account besides the van der Waals interactions, the angle, planar, dihedral as well as electrostatic interactions. Following the approach in [160], the MD aragonite tablets were constructed by repeating an aragonite unit cell. The considered three tablet geometries were minimised and equilibrated in the NPT (isothermal-isobaric) ensemble fore 10 ns at temperature 300 K and pressure 1.013 bar. The three cases did not show any remarkable changes, neither in the ordering of their structures nor in their cell dimensions. The last frames were employed to induce the cracks in the pulling simulations of the systems. For Mode I and Mode II simulations, no extension was applied to the periodic vector through the width *w*, which resulted in tablets of infinite length along *w*-direction. In fracture modelling of Mode III, the MD simulation boxes were extended along *w*, *l* and *h* of 40 nm, 20 nm and 10 nm, respectively. After that, the U-shaped notches were created by taking off atoms. To maintain the electroneutrality of the MD models, one calcium atom was removed from each carbonate group.

In the MD simulations, the simulation box, that includes the aragonite tablets subjected to pulling forces, was periodically repeated in the three dimensions with 2 fs as the time increments. 10 Å cutoff was considered for the van der Waals interactions. The Particle Mesh Ewald (PME) method [161] was considered for the long-range electrostatic interactions. For increasing the simulation time increment, we applied LINCS [162] for constraining all bond vibrations. Moreover, we implemented Nosé-Hoover [163,164] temperature coupling, where 0.1 ps as the coupling time constant. Figure 8 illustrates the pre-notched aragonite models with the related 3 loading cases and representation of the dimensions. In total, 12 systems were built of nearly 79,000 atoms. All of them were equilibrated in the NVT (canonical ensemble, with the preserved amount of substance N, volume V and temperature T) ensemble at temperature 300 K for 10 ns. To prevent the tablets from drifting, we restrained two cuboid volumes parallel to the (100) lattice plane, where each of the cuboid volumes includes the three outermost layers of CaCO${}_{3}$ atoms. After the equilibration step, we applied force-probe-MD (FPMD) simulations [165] to imitate an external force. In the FPMD modelling of the Modes I and II [43], pulling the restrained layers has succeeded via moving attached virtual spring with a constant of 1000 kJ mol${}^{-1}$ nm${}^{-2}$ and a constant velocity of 10 nm ns${}^{-1}$. In case of Mode III fracture, the bottom part of the geometry was restrained in all three directions using a virtual spring with constant of 1000 kJ mol${}^{-1}$ nm${}^{-2}$, whereas a fixed velocity of 10 nm ns${}^{-1}$ was assigned to the top boundary surface in the out-of-plane direction. The FPMD simulations continued until the onset of tablet fracture, which took place within ∼ 5 ns for Mode I, and ∼ 3 ns for Mode II, whereas for Mode III it was ∼ 7 ns. In this, four FPMD simulations were carried out each of the three modes.

**Figure 9:**
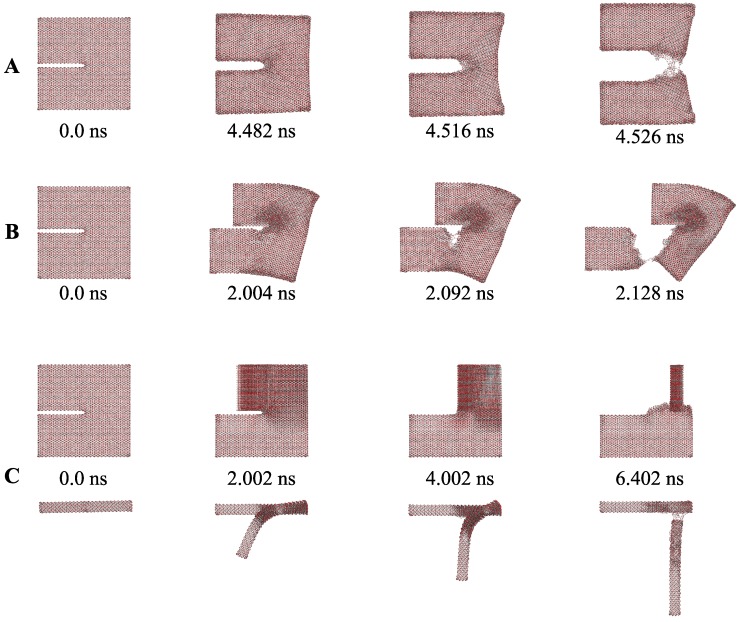
Fracturing of the aragonite tablet with initial flaws. The figures show the cracking under (**A**) Mode I, (**B**) Mode II, and (**C**) Mode III loading conditions.

Figure 9 illustrates the cracking processes at subsequent time steps of the CaCO${}_{3}$ tablet models. In Mode I (Figure 9A), a load with constant velocity was applied, where, except boundary atoms, no change in the atom arrangements was observed from 0.0 ns to 4.482 ns. In the next 0.040 ns, however, the crack started to initiate, and a brittle collapse took place. In Mode II case, bending induced an initial elastic deformation. However, after crack initiation, the failure occurred within 0.120 ns. In the case of out-of-plane loading, a progressive failure was observed.

**Figure 10:**
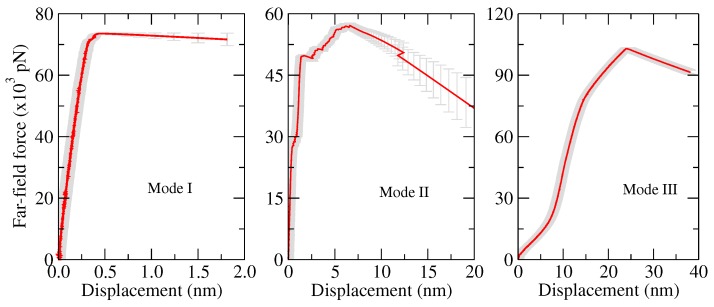
Force vs. displacement trajectories for the aragonite tablet models for Mode I, II and III. The red line represents the averaged curve related the different starting structures in MD simulations, and the gray shaded area is related to the standard error deviation.

Figure 10 presents the force–displacement curves of the MD simulations. From these curves, the stress–strain curves are derived. With regard to the material parameters, the slope of the stress–strain trajectory (initial slop) allowed to extract the Young’s modulus *E*, which was in the range of 110 to 144 GPa, agreeing with the values found in experimental and theoretical studies in the literature, see [26,27,166,167,168,169]. Using Griffith’s energy-based criteria, the energy release rate *G* is estimated from MD simulations. For Mode I MD simulations, the fracture onset of the aragonite tablet corresponded to a value *G* of 2.1 ±0.1 J/m${}^{2}$, which is in good agreement with values found in the literature [26,27,166,167,168]. We figure out that for Mode II and III, *G* values are 5.6 ± 0.8 and 6.0 ± 1.0, respectively. This allows the previously proposed bottom-up approach in Patil et al. [26] to be implemented for Mode II and III.

### 4.10. Silica Aerogel

Silica aerogels are classified as highly nanostructured solids. They are characterised by a very heterogeneous structure with a random arrangement of their atoms [35]. This material has a wide range of application areas, such as aerospace, automobile, and many more. Despite a wide range of current as well as potential applications of silica aerogels, one major concern is their possible collapse in a brittle manner. A careful study of the crack propagation and its influence on the resulting fracture strength, toughness and strain energy release rate remains to be examined. Therefore, recently, MD simulations were applied to study the fracture behaviour of silica aerogel, which showed an excellent performance in elucidating the crack onset and propagation in brittle materials [17].

**Figure 11:**
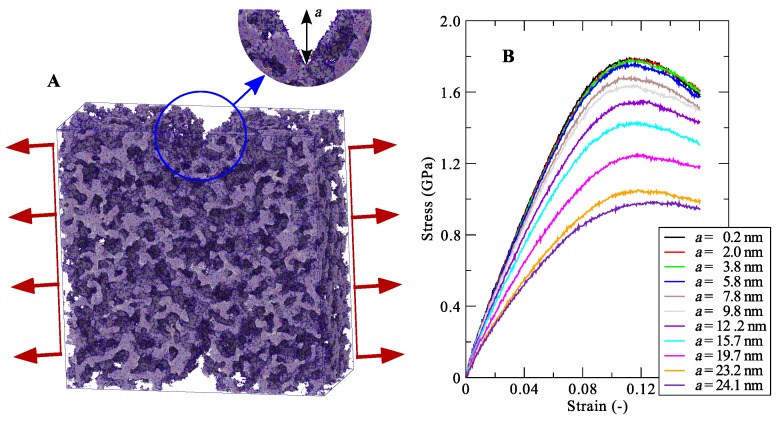
(**A**) Illustration of a MD model and initial notches of a silica aerogel sample with density of 645 kg m${}^{-3}$, where a (centre) refers to the notch depth. (**B**) Stress-strain trajectories extracted from MD simulations of silica aerogel models with a density of 1155 kg m${}^{-3}$ and various notch depths [17].

In our recent work, we used MD models to study silica aerogels subjected to tension-induced fracturing (see Figure 11) [17]. The focus was on understanding the brittle nature of the fracture and to estimate the fracture-related material parameters, e.g., the toughness, strain energy release rate, and the influences of the crack length on the fracture strength. For material densities ranging from 295 to 1155 kg m${}^{-3}$, we concluded that the toughness of the fracture and the strain energy release rate at crack onset increases with the crack length to height ratio. Additionally, these fracture attributes showed a power-law dependence on the aerogel material density with exponent values of 2.02 ± 0.05 for the toughness, whereas the exponent is estimated 1.16 ± 0.04 for the strain energy release rate [17]. The study allowed a more in-depth understanding of the cracking tendency and provided a mechanistic starting point for more reliable use of silica aerogels.

## 5. Conclusions

In the past two decades, a significant role has been played by MD simulations in developing an understanding of material behaviour and mechanical properties at the nanoscale. This comprehensive review elucidated the importance of MD simulations, which is a potent and valuable tool revealing many interesting hidden mechanisms and parameter correlations, which underlie the macroscopic behaviour. Moreover, because of the recent developments in the field of computer science, computers have become fast and inexpensive with significant memory capabilities, which is enabling many researchers to investigate the fracture processes of large-dimensions and complex materials.

Here, we summarised the crucial stages in the calculation of fracture mechanical properties from MD simulations using the macroscopic techniques such as Griffith’s criterion, J-Integral, crack tip opening displacement (CTOD) and other criteria. In particular, Griffith’s criterion is the most used theory to calculate the strain energy release rate, stress intensity factor and the dependence of fracture stress on crack depth. Recently, a multiscale modelling of brittle fracture has received enormous attention, wherein physically-motivated MD simulations are conducted to mimic quasi-static brittle crack propagation on the nanoscale and later correlated with macroscopic modelling of the fracture utilising the finite element technique. In this approach, the continuum parameters acquire an entire physical meaning. This bottom-up approach that entails of mechanics on the atomic and continuum levels can help multiscale modelling and reduce complexity, for example, the modelling and simulations of hierarchical-structured biomaterials and the tailoring of advanced materials.

The unique properties of highly brittle materials such as carbon nanotubes, graphene, silicon carbide, amorphous silica, calcium carbonate and silica aerogel, can be used for the current and many potential applications, and these materials can also provide new opportunities, e.g., reinforced composites. The success heavily relies on the selection of material for a particular application with having a fundamental understanding of its failure nature and fracture mechanical properties. In the present work, we studied the impact of crack length on SWCNTs, DWCNTs and graphene fracture using MD simulations. Moreover, the three modes of fracture studied on calcium carbonate, and the fracture properties were estimated from Griffith criteria.

Finally, the development of nanoscale fracture techniques is still under progress and is being consistently reviewed by eminent scholars from various disciplines. The key to success in this field is that the proposed methods have to be continuously reviewed by the researchers so that all the advantages, as well as drawbacks, can be addressed.

## Figures and Tables

**Table 1:** Milestones in MD simulations to study brittle fracture.

Authors and Year	MD System and Used Criteria	Brief Research Summary
Ashurst and Hoover (1976) [15]	First MD simulation to model fracture A small system of triangular lattice with 512 atoms Hooke’s law forces used for interaction of particles	A crack propagates through a crystal in a wide-spread damaged zone, and the obtained surface energy was more than that of a clean cleavage crack Studied fracture and crack propagation at microscopic scale
Decelis et al. (1983) [70]	MD simulations of alpha-iron and copper Griffith criterion used to estimate fracture properties	Brittle failure of alpha-iron along a cube plane at the critical Griffith value Brittle crack growth was prevented in copper due to blunting of a crack tip at a value less than $K_{I}$
Smith et al. (1989) [71]	MD simulations with EAM potential Studied embrittlement of hydrogen in Ni-based alloys	Studied the effect of hydrogen on Ni-based alloy’s fracture behaviour Hydrogen-embrittlement sensitivity is strongly connected to grain-boundary structure
Zhang et al. (1995) [72]	Copper f.c.c. structure under Mode I loading using MD simulations Analysis of ductile and brittle failure at the crack tip	Evaluation of different cleavages, and presence or absence dislocation emissions Studied the effect of atom configuration near the crack tip on the critical cleavage and nucleation of dislocations
Swiler et al. (1995) [73]	Non-equilibrium MD simulations of cristobalite and silica glass Used two- and three-body potentials	Glass shows a higher strain-rate dependence, while the crystal does to a lesser extent Analysis of coalescence of void volumes under Mode I loading
Abraham et al. (1997) [74]	Analysis of dynamic fracture for 2D notched solids Studied crack propagation under mixed mode loading	Studied crack branching and anisotropic effects of elasticity Showed that anisotropy plays a pivotal role in deciding the crack path
Holland and Marder (1998) [75]	Studied MD simulations of silicon using realistic two- and three-body potentials Investigated the crack velocity versus energy flux to the crack tip	Observed that up to a certain load, cracks are attracted to steady states Crack speed continues to increase smoothly until it reaches a velocity of 3586 m/s, at which juncture steady-state becomes unstable
Latapie and Farkas (2004) [76]	Investigated the influence of temperature, the fracture mechanisms and properties of nanocrystalline α-Fe Used EAM interatomic potential for the three samples, which were created using the Voronoi construction	In nanocrystalline α-Fe, observed that the overall fracture is due to an intragranular fracture combined with an intergranular fracture process The plastic deformation energy release and fracture resistance are found to increase with increasing temperature Observed that grain size decreases with increasing fracture toughness
Mielke et al. (2004) [77]	Calculations were performed using density functional theory (DFT), semiempirical methods and molecular mechanics Investigated how the vacancy defects play a vital role in the fracture of CNTs	For one- and two-atom vacancy defects, observed to reduce the failure stresses by ∼ 26 % and to reduce failure strains by factor of two For large defects, found that substantially reduce the failure stresses and failure strains
Zhou et al. (2008) [78]	Examined delamination at the interface of two different brittle materials Simulated crack propagation along the interface under mixed mode loading	Calculated the work done required for the separation of two materials in terms of loading mode mixity angle Derived an analytical expression for stress as a function of crack opening displacement
Kang et al. (2010) [79]	MD simulation of Si nanowires under Mode I loading Used of modified EAM potential	Concluded that the fracture depends on the temperature and nanowire diameter Shear failure was due to the nucleation of the flaws, and the cleavage fracture was caused by a crack on the surface
Terdalkar et al. (2010) [80]	Introduced method to study bond rotation and bond breaking Used Griffith’s fracture criteria	Studied rupture at the crack tip with localised high stresses Determined the minimum energy paths (MEPs) for the proposed fracture mechanism
Zhang et al. (2012) [81]	Studied fracture of graphene at the nanoscale under coupled in-plane opening and shear loading using MD simulations Used modified second-generation reactive empirical bond-order (REBO) potential [37] with a cutoff distance of 1.92 Å	Computed low toughness values indicate that at room temperature, strong graphene is absolutely brittle Torn zigzag edges are more kinetically and energetically preferable Crack propagation in graphene depends on local stresses, dynamic effects and edge energy
Tang et al. (2012) [82]	Studied the deformation and fracture behaviour of Si nanowires subjected to uniaxial tension and bending	Concluded that the structural properties and deformation behaviour of Si nanowire are firmly associated to wire diameter, loading conditions and stress states In uniaxial tension, Si nanowires show brittle failure, while under bending, they demonstrate considerable plasticity
Dewapriya et al. (2014) [24]	MD simulation of graphene sheets subjected to Mode I loading Studied of effects of nano-defects on its fracture strength	Computation of J-integral critical values, virial stress and Cauchy stress, critical stress intensity factor at different crack lengths Comparison of ultimate tensile strength calculated using MD simulation, Griffith’s theory and quantised fracture mechanics
Wang et al. (2015) [83]	Reported the crack propagation and fracture toughness of monolayer Molybdenum Disulfide (MoS${}_{2}$) sheets under mixed Modes I and II loading Used REBO potential for square MD model of side lengths of 110 Å and consisting of about 4700 atoms	Obtained $K_{c}$ values and crack propagation path of MoS${}_{2}$ depends on the crack edge chirality, crack tip structure and loading phase angle For all loading, cracks prefer to extend along a zigzag path for both armchair and zigzag cases
Patil et al. (2016) [26]	Performed brittle crack propagation in aragonite crystals Used Griffith’s criterion to compute energy release rate	Parameters calculated at the nanoscale were utilised in the continuum modelling A realistic physical interpretation to the phase-field modelling parameters was provided by all-atom simulations The combined approach can assist multiscale modelling of materials
Zhang et al.(2017) [84]	Studied crack propagation in Ni crystals Crystals subjected to Mode I loading	Investigated induced dislocations and role of the grain boundaries during crack propagation Observed the void formation and its further development into crack
Sumigawa et al. (2017) [85]	Used Griffith’s and classical fracture mechanics theory MD simulations to verify theoretical results	Correlated Griffith’s and classical fracture theories for fracture toughness (*K*) in nano-silicon Concluded that the *K* is inherent property and independent of the dimension of materials
Bao et al. (2018) [86]	Propagation of nanocracks in single layer MoS${}_{2}$ The dependence of the $\sigma_{f}$ on the *a* was established by Modified Griffith criterion	Studied strain rate, loading mode, temperature and crack type dependent brittle fracture behaviours of the single layer MoS${}_{2}$ using Showed that $\sigma_{f}$ decreases with increasing *a*
